# Determinants of Body Mass Index and Intelligence Quotient of Elementary School Children in Mountain Area of Nepal: An Explorative Study

**DOI:** 10.3390/children3010003

**Published:** 2016-02-03

**Authors:** Chhabi Ranabhat, Chun-Bae Kim, Myung Bae Park, Chang Soo Kim, Leila Freidoony

**Affiliations:** 1 Department of Preventive Medicine, Wonju College of Medicine, Yonsei University, 20 Ilsanro, WonjuCity, Gangwon-do 26426, Korea; parklove5004@naver.com (M.B.P.); l.freidoony@yonsei.ac.kr (L.F.); 2 Institute for Poverty Alleviation and International Development, Yonsei University, Yonseidae – gil, WonjuCity, Gangwon-do 26493, Korea; kimc@yonsei.ac.kr; 3 Health Science Foundations and Study Center, GPO – 44600 Kathmandu, Nepal; 4 Department of Business Administration, Yonsei University, 1 Yonseidae – gil , Wonju City, Gangwon-do 26493, Korea

**Keywords:** body mass index, intelligence quotient, growth, development

## Abstract

The physical growth and cognitive development of elementary school children are very crucial and this group is large in number but has little research dedicated to it. The physical growth and cognitive development of children occur simultaneously and can be measured by body mass index (BMI) and intelligence quotient (IQ). Previous studies could not sufficiently focus on both aspects. The aim of this study was to identify determinants of BMI and IQ of students in two elementary schools in the Humla district of Nepal. Two randomly selected elementary schools and all children available there (*n* = 173) participated in the study. BMI was calculated with the objective of proper measurement of height and weight of the children. Likewise, the updated universal nonverbal intelligence test (UNIT) was applied for IQ. Descriptive statistics, *t*-test, analysis of variance and multiple linear regressions were used when appropriate. Study findings showed that one-tenth of the children had grade 2 thinness (-2SD) and about one-third had poor IQ (<85). The age of the children (*p* < 0.05) and household economic status (*p* < 0.001) were significant for the BMI. Likewise, frequencies of illness in the previous year, mother’s education (*p* < 0.05) and father’s education (*p* < 0.001) were significant factors for the IQ score. More commonly, BMI and IQ scores were significantly lower in the ultra-poor group. Economic status and parent education are still major determinants of IQ and BMI in these students. Special programs and strategies should be launched to improve the poor ranking of IQ and BMI.

## 1. Background

Globally, about 668 million children are studying at the elementary school level, which is the largest proportion of the total population [1].The growth and development of these children progress simultaneously and are influenced by different factors [2]. Growth and development starts before infancy and continues up to the adolescent period [3]. Physical growth is the geometric growth of cells and can be directly observed. The growth of height, weight, and head and chest circumference are part of physical growth and increase vital signs as well as physiological ones [4]. Height and weight can be measured by body mass index (BMI). Likewise, child development can be observed in motor, emotional, social and cognitive developments [5,6]. Compared to physical growth, it is difficult to measure cognitive development. There are some special tests to measure different types of development of children, and intelligence quotient (IQ) testing is one method to measure cognitive development. Regarding the IQ of children, it is very difficult to predict and it can vary according to geographical location, age, gender, socio-economic factors [7], poor diet with high fat [8], and school environment [9].Currently, the problem of child obesity has been highlighted in developed countries but factors related to child growth and development in developing countries are less noticed [10,11,12]. Globally, more than one-third and 60% of families in developing countries are suffering from poor nutrition and this impact would reflect in the physical and cognitive development of children [13,14].

The prevalence of underweight children is four times higher (24%) in the rural areas of India in comparison with obese children [15]. In Makurdi, Nigeria, more than two-thirds of children had grade 2 thinness (BMI of less than 14) [16]. In Australia, however, only 5.3% of school children were reported as thin [17]. The BMI of children was average in those areas where parent education was good [18,19,20,21]. In Italy, it has been shown that people from a low socio-economic level are subject to low BMI and a higher risk of morbidity and mortality [21]. Likewise, the IQ of children is influenced by the consumption of several nutritional factors [22]. Low birth weight was also strongly associated with scores on the universal nonverbal intelligence test (UNIT), tests of executive function, and the movement assessment battery for children (MABC) of children in rural eastern Spain [23]. A study showed that there is a genetic influence from parents on children’s IQ in British and Dutch children [24]. Food that has been exposed to lead is associated witha lower child IQ in Europe [25]. Boss [26], and Robert Havighurst [27] have developed a theory of physical growth and cognitive development. It explains that individual factors such as age, gender, disease/illness, and household factors such as economics, food consumption pattern, education, school environment, as well as other factors related to gene and hormone composition, influence the BMI and IQ of children. Social inequality during the childhood period and the social context are equally responsible for child health status and overall development, including intelligence [28]. Studies in South Asia, including Indonesia, Malaysia, Thailand and Vietnam, reveal that undernourishment and poor IQ level should be explored together [29]. Nevertheless, most previous studies explored the situation of abnormal children rather than apparently healthy children, focusing on the social context and school environment in relation to physical growth and cognitive status especially in children from elementary school.

There are rare studies related to the BMI and IQ of children in developing countries and especially in remote areas. In those areas, there is a high proportion of poverty and food insecurity. Current studies focus more on genetic and disease-related factors affecting IQ and BMI rather than socio-economic factors. In Nepal, more than half of children less than three years of age [30] and about one-fifth of preliminary school children suffer from stunting and underweight, and the prevalence is higher in remote and ultra-poor families [31]. The National Demographic Health Survey (NDHS) 2011 revealed that in mountain districts of Nepal, including the Humla district, 29% of children below the age of five years were underweight, 8% were severely underweight, 11% were wasted, 3% were severely wasted, 41% were short for their age and 16% were severely stunted [32]. Humla is a very remote area of Nepal where about half of the population is under the poverty line. Food insecurity is extreme because there is less production of food and locally produced food is perceived as less nutritious and most of the people rely on imported food [33]. Transportation of food by air is expensive and is done by porters, which takes a long time. As a result, a proportion of nutrients is lost during the time of carrying and storage [34]. One-third of the children are not enrolled in school and the drop-out rate is also high [35]. Further, the environment is not appropriate for learning since most of the parents have had no formal education [36]. In the Humla district, 28.2% of children were undernourished, 8.8% were wasted and 22.4% were stunted among those under five years old [37], but the nutritional status and the IQ level of elementary school children are seldom investigated. The theory of physical growth and cognitive development and empirical findings indicate that socio-economic factors, parents’ education and food sources affect the BMI and IQ of school children. So, the aim of this study is to identify the determinants of BMI and IQ of elementary school children in relation to the household and school context in the Humla district of Nepal.

## 2. Experimental Section

### 2.1. Study Design and Sampling

**Figure 1:**
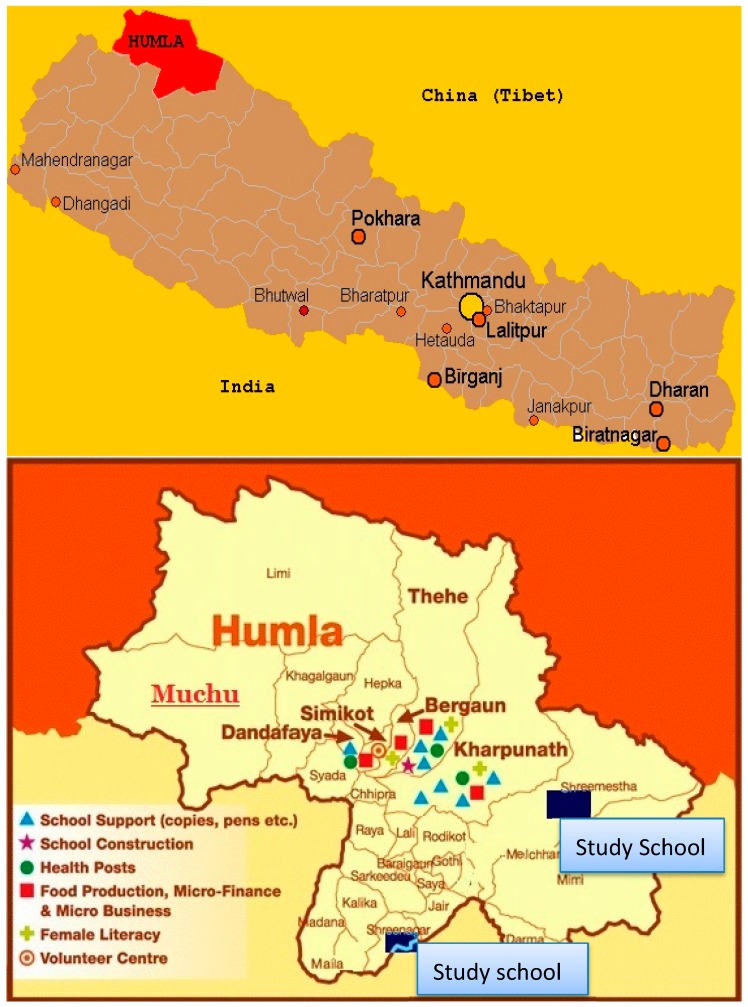
Area of study.

The study was a cross-sectional study based on elementary schools in the Humla district of Nepal. Humla was selected purposively as it represents the remote area of Nepal and other countries also, as shown in Figure 1 [38]. Two elementary schools (Shreenagar and Srimasta) were randomly selected and all children available at the schools were included in this study.

### 2.2. Study Setting and Measurement

Three sets of study tests were included in the study.

#### 2.2.1. Intelligence Quotient (IQ)

The universal nonverbal intelligence test (UNIT) [39] with six subsets of symbolic memory, cube design, spatial memory, analogic reasoning, object memory, and mazes was used with grade 5, 6, and 7 students. The six subsets included: (a) *symbolic memory*: ability to reproduce memories by showing sequences of symbols for 5 s; (b) *cube design*: ability to reconstruct the memorized geometric constructing designs using one-inch cube; (c) *spatial memory:* ability to reproduce the memorized patterns using green and black chips for 5 s; (d) *analogic reasoning:* ability to complete the analogy using incomplete analogy objects; (e) *object memory:* ability to memorize and indicate stimulus array using random pictorial arrays for 5 s; and (f) *mazes:* ability to correctly exit a maze using paper and pencil.

We prepared the set of tests having 160 items and those relevant events were adapted by Google images, some videos and graphic diagrams online. Those 160 test events were translated and prepared with local context so that students would easily understand them. For example, we prepared the incomplete analogies of local wooden cut pieces for analogic reasoning. Students’ total correct events among the total 160 IQ events were counted as their score. The process was closely monitored by teachers and researchers. The tests were administered to the children individually in a comfortable room that was well lit and free from noise. We scored all the students in the same way because such a test can be performed from five to 17 years age [40]. For the reliability, two observers closely observed the process, especially the time of each event. Moreover, video was taken of the test process for extra verification.

#### 2.2.2. BMI

The BMI was calculated based on height and weight for age of the children by anthropometric measurement (BMI = weight in kg/height in meters squared). Children were weighed using Seca digital scales which were validated with standard weights before actual weighing of the children commenced. The scales were placed on a hard flat surface. Children wearing only lightweight clothing (excluding shoes, belts, socks, watches and jackets) were weighed. Each child was measured twice and the measures compared to agree within 10 gram fluctuation. If the difference between the measures exceeded the tolerance limit (the degree to which the two measurements are close), the child was repositioned and re-measured a third time. The average of the two measures in closest agreement was recorded. For height measurement, the child stood with his/her back against the board, his/her heels, buttocks, shoulders and head touching a flat upright sliding head piece. The child’s legs were placed together with the knees and ankles brought together. Height was measured parallel to the occipital area of the standing child. The age of the children was based on the registration certificate available in school.

#### 2.2.3. Semi-Structure Questionnaire

A questionnaire was used to identify the parents’ education, food source, living and poverty status and obtained from the children’s home. Only the children’s parents who participated in our study were selected for interview. The concerned school teachers were appointed as field researchers who had taken the intensive training and completed the pre-test. The parents of the students, either father or mother or together, were the respondents to answer the semi-structured questionnaires. Class performance of each child was taken from individual records from related class teachers. In each question of questionnaire set, we made the options in numbers. Such numbers have been converted in a numeric code (1, 2, 3…) and measured accordingly.

### 2.3. Classification of BMI and IQ

The cut-off point for BMI was based on World Health Organization (WHO) standard growth reference of five to 19 years of age [41] which is used in most of the countries and research. There is no difference in average z-score on median score, -2SD (standard deviation) to +2SD in both genders. We made cut-off point by calculating mean of each age in -2SD, +2SD and median. So, the average of -2SD (second degree thinness) could be 15.3, median 16.8 and +2SD (overweight) is 24. Hence, the thinness was defined as <15.4, average 15.4–23.9, and high as ≥24 BMI *z*-score. IQ was categorized based on the book *Handbook of Psychological Assessment*, 2009 edition (<85 poor, 85–115 average and >115 good or smart) [42].

### 2.4. Characteristics of Variables

We classified the independent variables in three categories: (1) individual factors: age, gender, personality attitude, BMI, IQ and frequency of serious illness since last year; (2) school-related factors: grade, classroom performance, favorite subject and school attendance in last year; (3) family and social factors: economic status, father’s education, mother’s education and living status. Such variables were selected from previous research and local context that we described in background section. The main sources of food, living status, parents’ education, economic status of family were categorized according to Nepal Living Standard Survey 2011 [43]. The dependent variables, BMI and IQ, are related to physical growth and cognitive development.

### 2.5. Data Management

Before the data entry we prepared the range of response as 1, 2, 3… and we cross-checked by sorting each variable and comparing with results of the test. After this, we observed main variables for outliers by making the box plot and cross-checked for errors or highly skewed values. After verification we analyzed the data.

### 2.6. Data Analysis

The data were analyzed using SPSS version 21 (IBM SPSS Statistics for Windows, Version 21.0., Armonk, NY, USA) in two steps. In the first phase, the descriptive findings were arranged in frequency and percentage for categorical data, and mean and standard deviation for numerical data. Likewise, un-standardized association was set up with dependent and independent variables by independent *t*-test, and ANOVA. In the second phase, all of the predictors were entered into one model for BMI and same model was used for IQ using multiple linear regression models. However, for the predictors of age, BMI and IQ, we used the quadratic approach making the square of those variables because linear trends do not make sense for these variables. For the categorical variables, we ranked the variables making a reference group. The output was plotted in a table with constant (intercept) and beta coefficient for estimation. The grading of the student, economic status, father’s and mother’s education were converted into dummy variables making reference values of 0 and 1. The number of dummy variables was made as n-1 because one category was taken as reference group.

### 2.7. Research Equation Model

We entered all of the predictors (individual, school and family) into one model using multiple linear regression models at the same time. We ran one regression model for BMI and another for IQ. The equation model is shown as:

Y = b_1_x_1_ +b_2_x_1_^2^ + b_2_x_2_… α;

In variable square,Age, BMI and IQ had been entered into equation and other variables as linear regression model.

### 2.8. Validity and Reliability

The IQ test was taken from the standard principles of the universal nonverbal intelligence test with six basic subsets [39]. IQ test events were prepared with different images, video tutorials and test kits using web pages and available materials in school. All test events were translated and contextualized but the objective of each test event was not changed. Those events were verified with an education expert. The height and weight were measured by standard equipment and final measurement value was taken after two measurements. The questionnaires were used after a pre-test in similar household and intensive training was provided to the field researchers. Weighing scales were checked and validated with standard weights every day before actual weighing of the children commenced. Weighing machines were approved by Ministry of Health and Population (MoHP) of Nepal. Questionnaires were prepared based on the Nepal Living Standard Survey 2011. Likewise, the normality of data was verified by the observation of histogram and consistency of data was checked by Cronbach’s alpha in appropriate variables.

### 2.9. Ethical Consideration

We followed the national ethical guidelines by the ethical review board (ERB) for health research in Nepal 2011 [44] and formal approval was received from the district health and education office of the Humla district and selected schools, school children and their parents. Verbal consent was taken from school children and their parents for the study.

## 3. Results

### 3.1. Characteristics of Elementary School Students and Their Households

The study was performed with grade 5, 6 and 7 students from two elementary schools in the Humla district of Nepal. The mean ages of the grade 5, 6 and 7 students were 12.5 ± 1.5, 13.0 ± 1.3 and 14.2 ± 1.0, respectively. The mean age, height, weight and annual school attendance were 12.7 ±1.4 years, 125.5 ± 12.7 cm, 25.9 ± 6.6 kg, 96 ± 23.1 days, respectively. Likewise, BMI, IQ score and days affected by serious illness were 16.4 ± 3.5, 94.3 ± 10.8 and 0.6 ± 1.0 year. In our study, we found that 9.3% of the children had grade 2 thinness (BMI<15.4) and 30.6% had low IQ (<85). The main source of food, the current grade of the children and father’s education and economic category were significant for both BMI and IQ scores. Gender, personality attitude, favorite subjects, and living status were not significant for BMI or IQ. The students who were over 12 years of age had significantly higher BMI than children below 12 years (Table 1).

**Figure 2:**
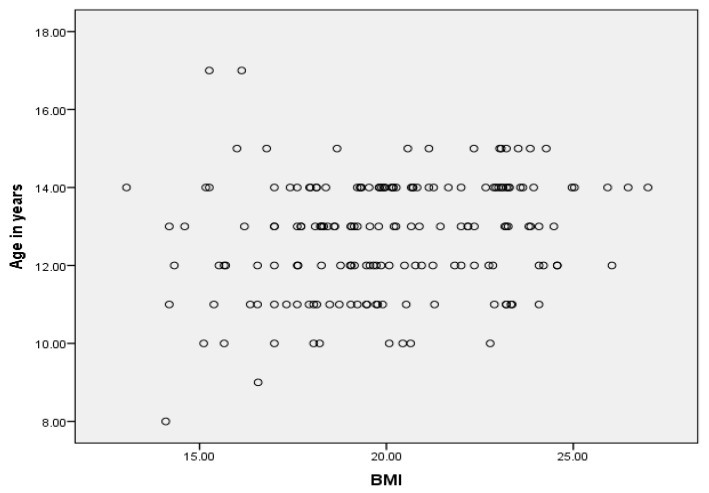

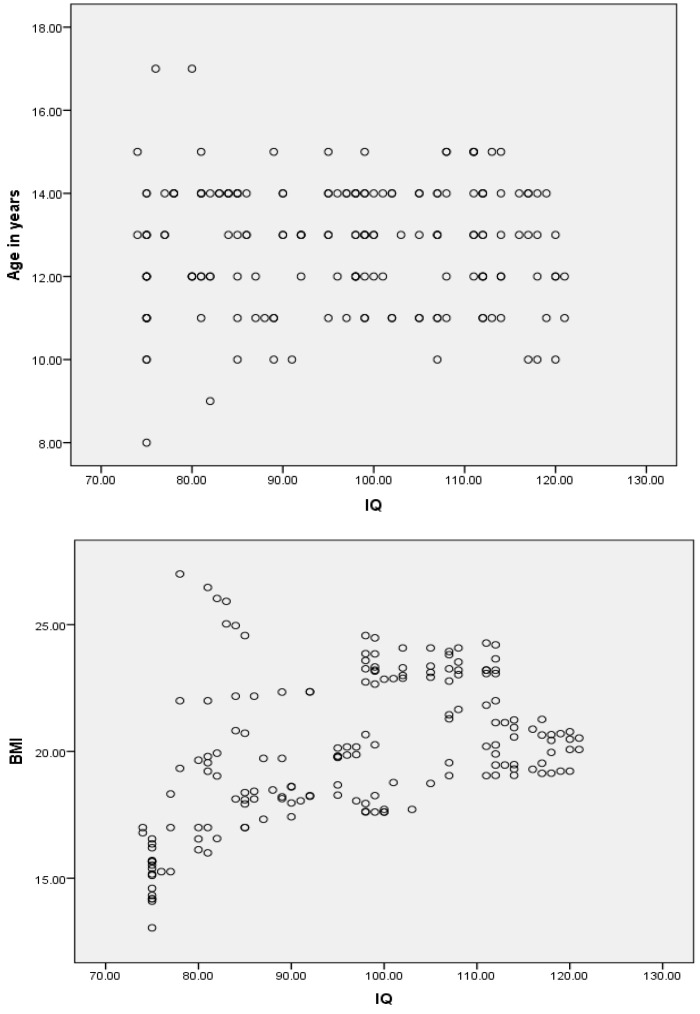
Association between age, BMI and IQ.

The Pearson correlation showed that there was a significant association between age with BMI and IQ score together (*p* < 0.01) and well as the frequency of serious illness per year with both BMI and IQ (*p* < 0.01), and a reciprocal relation between BMI and IQ (*p* < 0.05) without adjusting other variables. Among the categories of BMI, thin children had a significantly lower IQ than those with average and high BMI (*p* = 0.003), but among the IQ categories, BMI did not show a significant difference. The association between age and BMI and age and IQ was not in linear. Similarly, the association between BMI and IQ is also not proportional (Figure 2).

**Table 1:** Mean and standard deviation of BMI and IQ score with different predictors (*n* = 173).

Characteristics	Category	Frequency (%)	BMI		IQ Score	
			Mean ±SD	*p*Value	Mean ±SD	*p*Value
Gender	Boys	95 (54.9)	16.3 ± 3.1	0.221	95.2 ± 11.3	0.582
	Girls	78 (45.1)	16.6 ± 3.9		93.2 ± 10.2	
Age	Up to 12 years	109 (63.0)	19.6 ± 2.7	0.012	96.7 ± 14.6	0.795
	>12 years	64 (37.0)	20.7 ± 2.9		96.1 ± 13.6	
Personality Attitude	Faithful	6 (3.5)	17.8 ± 4.3	0.061	94.6 ± 9.4	0.312
	Active	95 (54.9)	15.9 ± 3.1		94.2 ± 10.8	
	Kind	45 (26.0)	16.4 ± 3.7		95.4 ± 11.1	
	Shy/Emotional	27 (15.6)	17.9 ± 3.8		92.8 ± 11.1	
Grade	5	75 (43.1)	14.4 ± 2.1	<0.001	92.0 ± 10.5	0.041
	6	56 (32.7)	16.9 ± 2.9		96.7 ± 10.8	
	7	42 (24.2)	19.4 ± 3.9		95.2 ± 10.8	
Classroom performance	Excellent	5 (2.9)	18.8 ± 4.5	0.078	96.0 ± 16.9	0.143
	Good	101 (58.4)	15.7 ± 3.1		93.7 ± 10.4	
	Average	60 (34.7)	17.1 ± 3.9		96.1 ± 10.9	
	Poor	7 (4.0)	18.0 ± 1.9		86.7 ± 9.1	
Favorite subject	Math	83 (48.0)	16.2 ± 3.4	0.122	94.0 ± 10.7	0.757
	Science	52 (30.0)	17.2 ± 3.7		95.3 ± 11.8	
	Social science	38 (22.0)	15.8 ± 3.2		93.8 ± 9.9	
Main source of food	Local	99 (57.6)	17.9 ± 3.3	<0.001	97.0 ± 9.6	0.041
	Imported	74 (42.4)	14.4 ± 2.7		90.6 ± 11.4	
Living with	Parents	8 (4.6)	16.8 ± 4.1	0.063	92.8 ± 10.0	0.701
	Parents and grandparents	95 (54.9)	15.9 ± 3.1		94.2 ± 10.8	
	Grandparents only	41 (23.6))	16.6 ± 3.8		95.6 ± 11.3	
	Others	29 (16.7)	17.8 ± 3.7		93.1 ± 10.8	
Father’s education	No formal education	8 (4.6)	12.8 ± 5.6	0.006	76.2 ± 3.8	<0.001
	Primary	62 (35.8)	15.6 ± 3.8		87.9 ± 7.9	
	Secondary	70 (40.5)	17.3 ± 3.1		95.8 ± 7.8	
	Higher education	33 (19.1)	17.0 ± 1.7		107.8 ± 5.0	
Mother’s education	No formal education	55 (31.7)	16.2 ± 4.4	0.34	86.7 ± 8.3	<0.001
	Primary	71 (41.1)	15.9 ± 3.5		92.5 ± 8.9	
	Secondary	40 (23.1)	17.5 ± 1.6		106.2 ± 5.2	
	Higher education	7 (4.1)	16.6 ± 2.0		105.0 ± 5.2	
Economic category	Ultra poor	18 (10.4)	12.1 ± 2.2	<0.001	82.3 ± 12.1	<0.001
	Poor	49 (28.3)	14.7 ± 2.0		93.1 ± 9.8	
	Relatively non poor	106 (61.3)	17.9 ± 3.3		96.9 ± 9.7	
BMI	Thin (<15.3)^a^	16 (9.3)			75.56 ± 1.54	0.003
	Average (15.4–23.9)^b^	121 (70.7)			98.00 ± 13.86	
	High (>24)^c^	36 (21.0)			100.75 ± 10.38	
IQ	Poor (<85)	53 (30.6)	19.5 ± 4.9	0.08		
	Average (85–115)	116 (67.1)	20.8 ± 2.6			
	Smart (>115)	4 (2.3)	21.3 ± 0.4			

BMI: body mass index; IQ: Intelligence quotient; SD: standard deviation.BMI *z*-score: ^a^-2SD; ^b^Median and ^c^+2SD, Cronbach’s alpha of all above variables is 0.695.

### 3.2. Regression Analysis

After the categorization of variables, regression analysis was performed to determine precisely which factors were significantly associated with the BMI and IQ of the children (Table 1). There was a significantly lower BMI for children in grades 5 and 6 in comparison with grade 7 and those children from poor and ultra-poor families in comparison with non-poor children (*p* < 0.001). For IQ, the predictors were quite different. There were significantly lower scores for IQ for children with less school attendances, frequent serious illness in the previous year and low parental education (*p* < 0.001) (Table 2).

**Table 2:** Determinants of BMI and IQ score by multiple linear regression mixed model.

Determinants	BMI	*p* Value	IQ Score	*p* Value
	Standardized Beta		Standardized Beta	
Intercept	17.1	<0.001	78.1	<0.001
**Individual Factors**				
Age in years	0.17	0.03	0.19	0.671
*Age*^2^	−0.22	0.03	−0.21	0.632
IQ	0.13	0.22		
*IQ*^2^	−0.10	0.01		
BMI			0.29	0.062
*BMI*^2^			−0.18	0.084
Frequency of serious illness	−0.15	0.11	−0.15	0.004
**School Factors**				
School attendance in a year			0.48	<0.001
Grade of student: 7 (*Ref.*)				
5	−0.18	0.04	−0.12	0.167
6	−0.13	0.02	−0.15	0.086
**Family and social factors**				
Economic status: Non poor *(Ref.)*				
Poor	−0.43	<0.001	−0.19	0.431
Ultra poor	−0.53	<0.001	−0.33	0.047
Father’s education: Higher (*Ref.*)				
No formal education	−0.01	0.83	−0.21	<0.001
Primary	−0.09	0.14	−0.20	0.004
Secondary	0.03	0.58	−0.12	0.045
Mother’s education: Higher *(Ref.)*				
No formal education			−0.44	<0.001
Primary			−0.28	0.002
Secondary			−0.12	0.162
Food type: Local food (*Ref.*)				
Imported food	0.06	0.30	−0.10	0.043
***R*^2^/Adjusted *R*^2^**		0.68/0.66		0.81/0.79
**F**		26.8		24.5
***p* value**		<0.001		<0.001

*Ref.*: reference group.

Table 2 shows estimated major determinants of BMI and IQ using a multiple linear regression model. The BMI would decrease by 0.18 (St. β) for grade 5 and 0.13 (St. β) for grade 6 students in comparison with grade 7 students and increased by age with 0.17. BMI would decrease for children from poor and ultra-poor families by 0.43 and 0.53 in comparison to those from non-poor families. The IQ score would increase by 0.48 with school attendance and decrease by 0.33 (St. β) in ultra-poor families in comparison to non-poor families. Likewise, the IQ score would decrease in those children whose parents had no formal education. By the nature of variables, the association between age, BMI and IQ could not be a linear relation. So, we include the squared terms in the regression model. The result indicates that BMI increases with decreasing speed as one gets older. The BMI and IQ have a similar relationship in the sense that these two variables are positively correlated but with a decreasing speed. Here, the association between BMI and IQ is borderline at the level of significance of 0.05; however, it is statistically significant at the 0.1.

## 4. Discussion

Our study revealed the major determinants of BMI and IQ of elementary school students who had no evidence of disease and deviation, living in the mountain area of Nepal. Poor socioeconomic status (SES) and parents’ education were the major determinants for lower BMI and IQ of children. Results showed that only 19% of fathers and 4% of mothers had higher education. More than one-third of households depended on imported food which is more expensive, less nutritious and directly affects child health [42,45]. More than one-third (Table 1) of the children’s families are struggling with poverty and such impacts showed in the BMI and IQ also. About one-tenth of the children had grade-2 thinness (BMI < 15.3) and low score for IQ (<85) compared to standard cut-off points. As we included in the background section, there is food insecurity and BMI and IQ are significantly lower for those who consumed imported food. Firstly, people cannot purchase imported food because it is expensive and the poverty level is high. Secondly, a proportion of nutrients are lost during the long time of transporting and storage [34]. The mothers who are mainly responsible for children’s food have insufficient knowledge about the importance of locally produced food because one-third of the mothers have no formal education. We observed the personality traits of the students also, but there was no association with BMI and IQ as found in a previous study by M. Bartels [46].

This study has two outcomes; BMI is related to physical growth and IQ scores to cognitive development. The BMI of children was significantly lower in grade 5 and 6 in comparison with grade 7 (not with the cut-off point) and by economic grading. The same situation has been found in poor and ultra-poor areas in contrast with non-poor households. Previous research based on these two perspectives are compared separately, starting with BMI. The students who used fast food outlets (some sort of imported food) had low BMI in Canada [47], a similar conclusion to our study. There was a positive association between age, grade and BMI during the childhood period [48,49] and the same trend showed in our study but the association is not linear (Table 2: Age^2^ ~ −0.22). Likewise, poor economic status of the household was found to be a good predictor of low BMI in all family members and especially in the children [50,51], and these findings match our study results. BMI was significantly lower in families of lower SES in comparison with middle and high economic status, and nutrition and quality of food were associated with BMI [52]. The height and weight of children were associated with age and poor economic status in rural Malaysia [53], Ethiopia [54] and Ghana [55]. Food insecurity and mothers’ inadequate education were responsible for low BMI in Nepal [56]. Those studies comfortably support our findings.

The determinants differed for IQ and BMI of children. IQ was inversely proportional to the frequency of serious illness and directly proportional to school attendance. On the other hand, higher education of the father and mother are equally relative to the children’s IQ score. Some previous findings support our result. Parent education, economic conditions and healthy diet were positively associated with IQ in an 11-year continuous study of children in a long-term cohort study in the UK [57]. Parent education and their cognitive ability has been found to be associated with the children’s IQ [58,59], the high IQ score of children being associated with high economic status in the household [60,61], and better school achievement [62,63]. These trends matched our results. A study in Calcutta showed poor cognitive development in six- to 12-year-old school children was significantly associated with poor economic status [64]. In another study, undernourishment and non-verbal IQ were significantly associated with low SES and parents’ education [29]. A report on children of Mauritius revealed that malnutrition at three years of age resulted in poor cognitive performance at 11 years of age, which signifies the impact of long-term poverty on children’s foundations [65]. A study done in Spain showed that children from low socioeconomic classes often experience low IQ and poor academic performance [66] and possess low scholastic achievement compared to medium or high SES children [67]. In the same way, SES is associated with physical fitness, cognitive, and socio-emotional outcomes in children in the US [68]. These findings closely match our results. Thus, for a better child IQ score, it is important to consider not only physical growth but also the parents’ contribution, regularity of attendance in school and less absenteeism due to diseases.

In bivariate analysis, a positive association between BMI and IQ was found, and this relationship is confirmed in the regression analysis, too. In the IQ score regression in Table 2, the coefficient for the BMI variable is positive (0.29) which is significant at the 10% level but not at the 5% level. In addition, Table 2 shows that the relationship is curvilinear with the coefficient of BMI^2^ of −0.18 and suggests that the IQ score increases with the BMI but with a decreasing speed. Previous studies have shown mixed results. A study in pre-school children in Iran by A.A.Tabrizin showed that there was a negative relation between IQ and BMI [69]. A systematic review by Satoshi Kanazawa in 2014 showed that obesity in children (BMI >22) was associated with low IQ and poor IQ is a risk for obesity (high BMI) [70]. There is not a consistent relationship between BMI and IQ in children under fiveyears of age [71]. Not only in children,however, but elders with a BMI of more than 25 had lower cognition function according to a study done in Spain [72]. However, some studies in the Netherlands concerning children at the age of six to 18 years revealed that intelligence was not associated with BMI [73,74]. A cohort study in New Zealand showed that people who had a higher BMI in childhood had lower IQ scores as adults but those who were obese as adults did not lose their IQ. Until now, there is no clear hypothesis between BMI and IQ; it may depend upon age, type of IQ test, genetics and other environmental factors and our findings are also unableto make a hypothesis.

Current studies are mostly focused on child obesity, but there is still child malnutrition (low BMI) due to food insecurity and poverty that not only affects the physical growth but also other factorsof child development. Our findings should alert policy-makers and researchers to pay attention to the physical growth and cognitive development of children in developing countries and remote areas. Large proportions of children in Africa and South Asia are still under-nourished and they could have poor IQ as well. Our findings indicated that lower BMI and higher BMI both are risks for poor IQ in children. BMI and different types of IQ tests should be observed in different age groups and those findings should guide special policy so that our future generations can be healthy and smart. Not only policy-makers but also future researchers need to explore other factors and pursue special types of research such as longitudinal and clinical trials because our study is only a cross-sectional one. There are some limitations to this study which must be considered. The sample size was quite small and we modified the universal non-verbal intelligence test (UNIT) for local context; however, we made the IQ events based on six subsets to make the results more valid. Being a cross-sectional study, some children were missed during the research time. Due to a large number of predictors in the regression model, it might not be powered appropriately. The records of the students were based on teachers’ observations rather than any standards. So, the findings may have some risk for generalization.

## 5. Conclusions

We found that parents’ education and household economic status were the strongest factors responsible for the BMI and IQ of the children. Policy-makers and program developers should be more concerned about it. Nutrition education for mothers on locally produced foods is strongly recommended. There are many factors responsible for the IQs of children. School regularity, parents’ education and parents’ attention for the children’s learning will improve the overall education of the child, including the IQ. Along with those activities, poverty alleviation packages and promotion of local food by special program interventions are crucial.
